# Characteristics of youth sexual and reproductive health and risky behaviors in two rural provinces of Cambodia

**DOI:** 10.1186/s12978-015-0052-5

**Published:** 2015-09-07

**Authors:** Jaime R Lopez, Pamela E Mukaire, Ronald H Mataya

**Affiliations:** School of Public Health, Department of Global Health, Loma Linda University, Loma Linda, California 92354 USA; School of Public Health, Department of Epidemiology, Biostatistics, and Population Medicine, Loma Linda University, Loma Linda, CA USA; School of Public Health, Department of Health Promotion and Education, Loma Linda University, Loma Linda, CA USA

**Keywords:** Cambodia, Rural youth, Sexual and reproductive health, Risky behaviors

## Abstract

**Background:**

The global number of youths has risen with a majority living in Southeast Asia. In Cambodia, rural youths often face difficult barriers to health, which include lack of sexual and reproductive health knowledge, information, and services. Risky behaviors are a threat to the health of many young people in Cambodia.

**Methods:**

We studied a sample of 300 youths to describe sexual and reproductive health characteristics and risky behaviors in two rural provinces of Cambodia. Using a multi-staged sampling method, 30 villages were selected for interviewing. A peer-to-peer interviewing criterion was used that matched interviewer to interviewee based upon sex. Logistic regression models were used to compare risk between sexes and assess for associations between reproductive health variables, gender, youth attitudes, and risky youth social behaviors.

**Results:**

A majority (90 %) stated that a boy or girl should defer sex till marriage. A majority of youths (92 %) also reported that they may or definitely will seek sexual and reproductive health services in the future. About 5.4 % of youth had a prior sexual experience. Only 6.7 % of youth reported having they traveled to a local health center, hospital or clinic to seek healthcare for a reproductive health problem. Overall, 27 % reported alcohol use in prior 30 days. Relative to girls, boys were more likely to report alcohol use, going out late at night with friends, gambling, pornography use, gambling, and practicing risky behaviors with peers. Living with both parents and current school enrollment, had limited impact on rural youth’s individual and social behaviors.

**Conclusion:**

Although there are favorable findings compatible with traditional Cambodian values and beliefs, the youth in this study are challenged with alcohol use, practicing risky behaviors with peers, and low condom use. Findings have implications for practice and policy to prevent substance abuse and improve outcomes for substance use, sexual and reproductive health.

## Introduction

The world’s adolescent population has increased to the highest level in recent history.

In 2012, the world had 1.6 billion persons aged 12–24 year, 721 million of which were adolescents aged 12–17 years, and 850 million were youth aged 18–24 years [1]. In the Asia Pacific region, youth comprise about 712 million people [2] with millions of these young people often lacking adequate education to allow for adequate sustainability [3, 4]. 

Cambodia has the youngest population in Southeast Asia, with 22 % of the population comprising of young people is between 15 and 24 years of age [5, 6]. It is estimated that there will be about 4 million people under the age of 24 year by 2015 [7, 8]. In 2008, the general population census reported that Cambodian youth (10–24 years) represented over 34 % of the national population [9]. According to a 2009 report by the Population Council, young people face many concerns in terms of sexual and reproductive health [10] such as sexually transmitted diseases, unwanted pregnancies, unsafe abortion, and HIV and AIDS. These health concerns are exacerbated by the lack of information, knowledge, services, and poor education (i.e. low school enrollment, high dropout rates, and high repetition rates) as reported by the World Health Organization [11, 12].

Globally, research of adolescent sexual and reproductive health has been concentrated in western countries. In Cambodia, sexual and reproductive health is a major concern and few investigations have been completed [13–15]. Young people are increasingly exposed to new cultural trends, and yet their parents have difficulty sharing information on sexuality. These issues are a sensitive issue in Khmer culture and especially for the rural young people. Many times the rural youth are ill-informed, and with no parental involvement due to a lack of sexual health knowledge and life-skill training. The lack of valid health information such as HIV transmission and others has led to misconceptions about the nature of the reproductive health diseases, resulting in increased risky health behaviors for this age group [16].

The youth seldom delay marriage past their mid-20s. In Cambodia, the estimated age at first marriage is 23.3 years for girls and 25.5 for boys [17]. Parity is often initiated early with about 23 % of young married girls having their first child by the age of 19 years. In 2004, the Cambodian National Youth Risk Behavior Survey stated that 37 % of girls aged between 15 and 24 years reported that they had an unmet need for family planning, with only about 13 % reporting using contraceptive methods. National rates of HIV infection has decreased since 2001, moreover, it is reported that the bulk of infections occur between 15 and 24 years [18–20]. The reported incidence of HIV infection for this age group, was about 0.2 % [9, 21].

Some young Cambodian boys and girls engage in risky behaviors at a very young age. For example young people out of school reported having sex early in adolescence. Among those who reported using alcohol, many reported consuming alcohol at the age of 12 years [22]. In 2004, the United Nations Children’s Fund and United Nations Educational, Scientific and Cultural Organization conducted the first survey to assess the risk behavior of Cambodian youth between the ages of 11 and 18 years, and concluded the following: 1) 50 % or more of young people were illiterate, 2) those in school were less at risk as compared to out-of-school youth, 3) one-third of sexually active youths never wore a condom and of these 24 % were unaware about sexually transmitted diseases, 4) 90 % knew about avoidance of HIV/AIDS but only 53 % were educated in HIV transmission and infection, 5) 48 % of youths in rural areas lacked primary education, 6) 33 % of youths knew someone who had participated in gang rapes, 7) 40 % youths who engaged in premarital sex consumed alcohol [23].

For rural Cambodian youth, sexual and reproductive health is a major public health concern. Evidence suggests that healthcare services available to youth do not always encompass sexual reproductive health services; and that, services offered to youth are not differentiated from those of infants or adults [24]. These approaches fail to acknowledge increasing trends in delay of marriage among youth and married couples that often delaying their childbearing practices [24, 25]. Key changes in norms which affect all societies, have expanded the gap between puberty, –marriage and childbearing [26]. To the best of our knowledge, we are not aware of any prior studies examining rural youth sexual and reproductive health. We used data collected from the 2009 Rural Cambodian Youth Sexual and Reproductive Health project [27] to examine sexual and reproductive health practices among youths of two rural provinces. These rural regions of Cambodia include communities of ethnic hill tribes and historical sites such as temples from the pre-Angkorian era. Our study objectives were, 1) to examine reproductive health attitudes, beliefs, and values of rural youths, and 2) to examine variables associated with youth sexual and reproductive health attitudes and risky behaviors in rural youths.

## Methodology

### Sampling and eligibility

From March through April 2009, we conducted this study in rural areas Kampong Thom and Preah Vihear provinces. We used a multi-stage sampling method to recruit local rural youths for our survey. In the preliminary stages, in each province, we randomly selected a local district, commune and village. In each village, the local leadership was consulted and afterwards the central location of the village was determined. Next, a systematic approach was used to select households for recruitment by selecting each third home from the central location. The study population consisted of 300 rural youths aged 10 to 24 years, from 300 households that comprised 30 villages. In each of the selected villages, 10 households were selected and 5 boys and girls were invited to participate. If a household did not include a youth or declined another household was selected. Our eligibility criteria included: 1) youth aged 10 to 24 years, 2) the consent from parent or guardian and each youth after explanation of study purpose, risks and benefits, 3) youth’s permanent residence of the household selected, and 4) the ability to understand and respond to survey questions.

### Surveyors

For interviewing, fifteen surveyors and supervisors were recruited and trained in a two-day workshop focused on the administration of the survey questionnaire. Surveyors from the University of Phenom Penh, Department of Sociology, completed door-to-door interviewing, that included an interviewer-interviewee criterion that matched boy-to-boy and girl-to-girl. Most of our surveyors were from rural areas of Cambodia and had prior experience working in rural communities. All interviewing was completed in a private location inside or outside the home where each youth was assured privacy. This interviewing, criterion resulted in a 62.6 % response rate. We obtained ethics approval for our study from the Ministry of Health, Ethics Committee for Human Research, Phenom Penh, Royal Kingdom of Cambodia [28].

### Survey questionnaire

We constructed our survey questionnaire in English and then translated it into the national Khmer language using our surveyors from the University of Phenom Phen. We made some initial changes and modifications to the survey questions based on review by local public health professionals, and our survey team in Cambodia. Prior to data collection, we pilot tested our survey questionnaire and modified the survey to increase comprehension of questions. We used two separate surveyors to translate the questionnaire into Khmer and back into English to check for any inconsistencies, and make changes to the final survey questionnaire. Data was entered into Microsoft Access ® 2007 software using a double-entry method and we used Epi info Data Compare ® version 3.5 for identifying inconsistencies.

We collected data regarding demographics, socioeconomics, and migratory practices. We categorized demographic variables that included religious affiliation, marital status and parental residence. To measure socioeconomics, we categorized current grade level (using the Cambodian educational system), occupation, and income earner (s) of the household. An additional item asked to each rural youth was, “who controls your income?” to measure independence level and decision making ability.

The need for income supplementation was assessed by including questions regarding the need to migrate for income, and if their household income was enough. We asked each youth if they ever migrated, and if, where they migrated. We collected information about migratory occupation and abuse, and if any, abuse type.

To measure the adherence to traditional Cambodian (Buddhist) rural youth values, beliefs, and attitudes toward sexual activity, we included survey questions regarding these characteristics. Our survey questionnaire included questions about the ideal status of a youth (unmarried or married) and sexual activity for both sexes. We assessed beliefs about sexual activity by including a survey item that asks, “What is the ideal age for a boy or girl to have sex?” Data of youth attitudes of sexual activity were measured by including items in the questionnaire that included, “Do you ever want to marry someone?” For attitudes toward sexual activity in their remaining adolescence was asked, “Do you expect to have none, one or more sexual partners?” Regarding attitude toward seeking sexual reproductive health services in the future, we asked, “Do you plan to see a provider?” These items have been associated with negative sexual reproductive health outcomes [29].

Romantic relationships in young people often prepare them for future marriage and family for Cambodian rural youth. To measure these characteristics, we included questions in our survey that asked each rural youth if they were ever or currently in a relationship, their commitment level, age at first sex (debut) and type (free choice or forced), and if they presently “feel ready to cause a pregnancy.” Ready to cause a pregnancy is a risk indicator of sexually transmitted diseases HIV and other risky behaviors [29].

We collected information about the rural youth’s healthcare seeking behaviors. We categorized data by provider type that was accessed in prior 6 months. We asked the reason for requiring services to assess the level of services and available information. Categories of healthcare service provider included a hospital, health center, clinic, local pharmacy, traditional healer, shop keeper, or a village support group (VHSG). We asked reasons for seeking care that included curative (i.e. infections), prevention (non-reproductive health), and prevention-specific sexual reproductive health services. Trust is a major reason for seeking care among the youth. Confidence and confidentiality are important to each youth. In this regard, we asked provider type to assess which type the youth had the most confidence in; from a list that included a physician, nurse, pharmacist, village shopkeeper, peer counselor, and a traditional healer.

To measure alcohol use and risky behavior in rural youth, our survey categorized alcohol use and frequency of use, in prior 30 days. Furthermore, youths were asked if they ever had sex under the influence of alcohol. We included survey items asking the youth if they ever tried drugs, frequency of drug use, and if they ever had sex under the influence of drugs. Youth risky practices were categorized under out late at night with friends, gambling, pornography use, and practice all risky behaviors. Condom use was assessed by asking youth if they had sex with alcohol or drug use, and if they used a condom.

### Data analysis

Our survey data was analyzed with SAS 9.4 for Windows (SAS Institute Inc., Cary, NC) allowing for survey design and nonresponse in both estimates, and corresponding standard errors (SE). We constructed survey weights that were derived from the 2008 Cambodia census data. Continuous variables were analyzed using PROC SURVEYMEANS and reported with the corresponding standard error of the mean (SEM). We used a non-parametric Mann–Whitney test to assess for significant median difference of sexual reproductive health variables. PROC SURVEYFREQ was used to analyze categorical variables and included a test for association using Rao-Scott Chi Square test. Married youths were only included in the analysis of socio-demographics. All other analyses included unmarried rural youths. All tests were two sided with significance level at p < .05.

We conducted an analysis to compare in individual and social behaviors between unmarried boys and girls. The individual behaviors included alcohol and drug use. Social behaviors included going out late night with friends, pornography use, gambling, and practicing risky behavior with peers. We used the girl category as the reference. We report odds ratios with 95 % confidence intervals to assess risk between the sexes.

We used a logistic procedure to assess the effects of current school enrollment and living with both parents on unmarried youth attitudes and risky behaviors. Youth attitudes included seeking sexual reproductive health services, and feeling ready to cause a pregnancy. Risky behaviors included out late at night with friends, gambling, pornography use, practicing risky behaviors. We report crude odds ratios with 95 % confidence intervals and estimates adjusted for age and sex.

## Results

### Socio-demographics

A summary of socio-demographics of the study population is shown in Table 1. The mean age of the study population was 17 years (SD = 0.1). Between the sexes, boys were older than girls. We assessed age categories and found that 40 % of rural youths were between 15–19 years and boys represented the older categories and girls younger categories. There were twice as many young girls compared to boys. Youth demographics included the Buddhist religious tradition (98.7 %), single marital status (87.7 %), and were living with both parents (84.3 %). Youths reported their current Cambodian school 3–6 grade level (42.2 %) and lower secondary 7–9 grade level (35.7 %).

**Table 1:** Demographics, socioeconomics, and migratory practices

	All		Boys		Girls		p-value
	n	% (SE)	n	% (SE)	n	% (SE)	
Demographics							
Age in years (mean ± SEM)		17.0 ± 0.1		17.9 ± 0.1		16.2 ± 0.1	t = 9.2, p < .0001
Age categories							*X* ^2^ = 595.0, p < .0001
10-14 years	90	30.2 (0.4)	30	20.5 (0.8)	60	40.0 (0.2)	
15-19 years	122	40.5 (0.3)	62	41.0 (0.3)	60	40.0 (0.2)	
20-24 years	88	29.2 (0.5)	58	38.4 (1.1)	30	20.0 (0.7)	
Religious Affiliation							*X* ^2^ = 0.002, p = 0.9
Buddhist	296	98.7 (0.6)	148	98.7 (0.9)	148	98.7 (0.9)	
Christian	4	1.3 (0.5)	2	1.3 (0.7)	2	1.3 (0.8)	
Marital status							*X* ^2^ = 2.1, p = 0.2
Single	263	87.7 (1.3)	136	90.7 (2.2)	127	84.8 (2.6)	
Married/Divorced/Widowed	37	12.3 (1.4)	14	9.3 (2.3)	23	15.2 (2.5)	
Living with both parents							*X* ^2^ = 1.3, p = 0.2
Yes	252	84.3 (2.1)	129	86.7 (2.7)	123	82.0 (3.0)	
No	47	15.7 (2.2)	20	13.3 (2.5)	27	18.0 (3.1)	
Socioeconomics							
Current grade level							*X* ^2^ = 2.8, p = 0.2
Grade 3-6	74	42.2 (3.8)	30	36.3 (4.7)	44	46.9 (5.6)	
Lower secondary 7 - 9	62	35.7 (3.6)	30	36.9 (4.7)	32	34.8 (4.6)	
Secondary 10 - 12	39	22.1 (4.6)	22	26.8 (6.5)	17	18.3 (5.7)	
Current occupation							*X* ^2^ = 23.8,p < .0001
Student	188	71.8 (3.3)	83	61.0 (4.3)	105	82.6 (4.3)	
Service oriented or laborer	62	23.3 (3.4)	48	35.3 (4.4)	14	11.1 (3.1)	
Goods seller or other	13	4.9 (1.4)	5	3.5 (1.4)	8	6.3 (2.5)	
Who earns income for your household?							*X* ^2^ = 3.1, p = 0.05
Myself	23	7.9 (1.6)	16	10.7 (2.4)	7	4.7 (2.0)	
My father/mother/sister/brother	266	92.1 (1.5)	134	89.3 (2.3)	132	95.3 (5.0)	
Who controls your earned income?							*X* ^2^ = 0.7, p = 0.4
Me	6	9.1 (3.1)	3	6.9 (3.8)	3	13.1 (6.2)	
Someone else	61	90.8 (3.2)	41	93.1 (3.9)	20	86.9 (6.3)	
Does your household have enough income?							*X* ^2^ = 3.9, p = 0.04
Yes	138	46.6 (3.1)	79	53.3 (5.4)	59	39.8 (4.1)	
No	159	53.3 (3.0)	70	46.6 (4.5)	89	60.1 (4.0)	
Migratory practices							
Ever migrated for income							*X* ^2^ = 0.01, p = 0.9
Yes	63	21.0 (2.0)	32	21.3 (3.4)	31	20.8 (3.1)	
No	235	78.9 (2.1)	118	78.7 (3.5)	117	79.2 (3.0)	
Migratory destination							*X* ^2^ = 7.2, p = 0.02
Phnom Penh City	30	48.8 (6.9)	10	33.1 (9.1)	20	62.3 (10.0)	
Inside their province	21	34.8 (6.4)	14	49.6 (8.5)	6	7 (7.2)	
Outside their province	10	16.4 (5.5)	5	17.1 (6.5)	5	5 (7.5)	
Migratory occupation							*X* ^2^ = 0.4, p = 0.5
Manual skilled job/Driver	59	95.2 (2.5)	30	96.8 (3.1)	29	93.6 (4.2)	
Service orientated occupations	3	4.8 (2.6)	1	3.1 (3.0)	2	6.4 (4.2)	
Migratory occupational abuse							*X* ^2^ = 2.3, p = 0.1
Yes	11	24.7 (5.9)	9	33.5 (10.1)	2	11.5 (8.1)	
No	33	75.3 (5.8)	18	66.4 (10.0)	15	88.4 (8.2)	
Migratory occupational abuse type							*X* ^2^ = 1.3, p = 0.5
Discrimination	3	14.8 (7.4)	1	9.5 (9.8)	2	20 (13.3)	
Fraud the salary	7	34.4 (12.6)	5	49.2 (22.6)	2	20 (13.3)	
Other	10	50.7 (12.2)	4	41.2 (23.2)	6	60 (13.0)	

Youths represent a majority of the Cambodian workforce. We found that 71.8 % reported their current occupation as a student, with more girls (82.6 %) reporting than boys (61 %). About 23.3 % reported working in a service orientated or laborer occupation. There was 3 times more boys (35.3 %) than girls (11.1 %) reporting this occupation. Household income was earned by someone else other than the youth was 92.1 %. More boys (10.7 %) reported earning an income than girls (4.7 %). Similarly, 90.8 % youths reported that someone else controlled their earned income with more girls (13.1 %) than boys (6.9 %) reporting control of their income. We noted that more girls (60 %) reported that their household income was not enough to sustain their households. Low minimum wages and no employment opportunities are frequent reasons.

We found that about 1 in 5 (or 21 %) of rural youth reported they migrated for earned income. There was no difference between sexes. Phenom Phen was the most frequently reported destination, followed by a center inside their province and outside their province. More girls migrated to Phenom Phen (62.3 %) while boys migrated to a center within their province (49.6 %). A majority of youths reported their occupation during migration as manual-skilled job or a motordup (i.e. taxi) driver (95.2 %). Migratory occupational abuse was reported by more boys (33.5 %) than girls (11.5 %), and included discrimination and fraud of the salary (i.e. incomplete compensation for work).

### Sexual and reproductive health characteristics

Table 2 shows a summary of sexual and reproductive health values, beliefs, attitudes and relational characteristics of rural youths. We found that boys and girls equally value the status of sex after marriage for a girl (93.9 %) and boy (90.1 %). When asked about the age that a boy or girl can have sex; girls reported a younger age (18 years) than boys (20 years). A majority of youths (79.8 %) reported the desire for future marriage with more girls (89.6 %) reporting than boys (72.5 %). We asked about attitude toward future sexual partners and found 78.3 % expect to have at least one sexual partner in their remaining adolescence. More girls (83 %) reported expecting to have at least one sexual partner than boys (73.8 %). Among the rural youths, there was about 18.2 % reported a prior relationship with a boy or girl. There were more boys (21.1 %) than girls (15.2 %) who reported a prior relationship. More boys (16.7 %) were in a current relationship than girls (10.5 %). Youths relationship commitment level was “very committed” in boys (87.2 %) than in girls (53.3 %). Only a small number (n = 14) of youth reported their age at first sex (debute) as being 19 years of age. First sex was reported to be a free choice and not forced by 85.2 % of youths; however, this represents only fourteen youths. There was about 19.2 % of youths reporting that they are currently ready to cause a pregnancy and might be at risk of negative reproductive health outcomes (i.e. unwanted pregnancy).

**Table 2:** Sexual reproductive health values, beliefs, attitudes, and relational characteristics

	All		Boys		Girls		p-value
	n	% (SE)	n	% (SE)	n	% (SE)	
Sexual reproductive health values							
What is the ideal status for girl to have sex?							*X* ^2^ = 0.01, p = 0.9
Before marriage	15	5.7 (1.5)	8	5.9 (2.1)	7	5.5 (1.9)	
After marriage	245	93.9 (1.6)	126	94.1 (2.1)	119	93.6 (2.0)	
What is the ideal status for boy to have sex?							*X* ^2^ = 0.5, p = 0.4
Before marriage	25	9.5 (1.5)	15	10.9 (2.5)	10	7.9 (2.4)	
After marriage	237	90.1 (1.6)	121	89.0 (2.5)	116	91.2 (2.4)	
Sexual reproductive health beliefs							
What is the ideal age for a girl to have sex? (median and range)	263	18 (11–25)	136	18 (11–25)	127	18 (15–25)	z = 2.8^a^, p = 0.004
What is the ideal age for a boy to have sex? (median and range)	263	20 (10–30)	136	20 (10–30)	127	20 (14–30)	z = 3.8^a^, p = .0001
Sexual reproductive health attitudes							
Do you ever want to marry someone?							*X* ^2^ = 5.7, p = 0.01
Yes	145	79.8 (2.3)	78	72.5 (3.9)	67	89.6 (3.5)	
No	37	20.2 (2.1)	29	27.5 (4.0)	8	10.4 (3.4)	
Attitudes towards future sex in remaining adolescence							-
Expect no sex partners	42	16.1 (2.3)	24	17.4 (3.2)	18	14.6 (3.3)	
Expect one sex partner	203	78.3 (2.4)	100	73.8 (3.8)	103	83.0 (3.2)	
Expect more than one sex partner one at a time	10	3.7 (1.2)	7	5.1 (2.3)	3	2.4 (1.3)	
Expect more than one sex partner more than one at a time	5	1.9 (0.9)	5	3.6 (1.8)	-	-	
Attitudes towards seeking SRH^b^ services in future							*X* ^2^ = 7.2, p = 0.02
I do not plan to see a provider	19	7.2 (1.7)	9	6.6 (2.3)	10	7.8 (2.8)	
I may or definitely will see a provider	243	92.8 (1.8)	127	93.3 (2.1)	116	92.2 (2.7)	
Relational characteristics							
Ever had sex?							*X* ^2^ = 5.8, p = 0.01
Yes	14	5.4 (1.2)	11	8.4 (2.0)	3	2.4 (1.3)	
No	243	94.6 (0.2)	120	91.6 (0.9)	123	97.6 (0.4)	
Ever had a boy/girlfriend or partner							*X* ^2^ = 1.9, p = 0.2
Yes	48	18.2 (2.4)	29	21.1 (3.2)	19	15.2 (3.4)	
No	215	81.8 (2.5)	107	78.8 (3.1)	108	84.7 (3.5)	
Currently has boy/girlfriend or partner							*X* ^2^ = 4.2, p = 0.04
Yes	36	13.6 (2.1)	23	16.7 (2.5)	13	10.5 (2.6)	
No	227	86.3 (2.2)	113	83.3 (2.6)	114	89.5 (2.7)	
Commitment level in current relationship							*X* ^2^ = 4.9, p = 0.03
Very committed and want to marry	28	74.4 (5.7)	21	87.2 (7.1)	7	53.3 (13.2)	
Not committed and do not want to marry	9	25.5 (4.6)	3	12.8 (7.2)	6	46.7 (13.2)	
Age at sexual debut (median and range)	14	19 (17–24)	11	19 (17–24)	3	18 (15–21)	z = 1.2^a^, p = 0.2
First sex type							*X* ^2^ = 0.8, p = 0.3
Free choice and wanted	12	85.2 (10.6)	10	91.2 (8.8)	2	64.5 (0.0)	
Forced or coercive	2	14.7 (11.5)	1	8.7 (8.7)	1	35.5 (0.0)	
Do you feel you are ready to cause a pregnancy now?							*X* ^2^ = 0.9, p = 0.4
Yes	35	19.2 (2.7)	23	21.5 (3.7)	12	16.2 (4.1)	
No	146	80.8 (2.7)	83	78.5 (3.7)	63	83.8 (4.1)	

^a^Mann-Whitney test for difference in medians, ^b^Sexual Reproductive Health

### Health seeking behaviors

In regards to health seeking behaviors, we found that 72.5 % of rural youths reported visiting a medical facility such as a local health center, hospital, clinic or pharmacy (Table 3). Boys were more likely to report vising a local health center facility than girls. More girls (42.2 %) reported to visit a non-medical facility for health services in the prior 6 months, to include a traditional healer, shop keeper, or village support group.

**Table 3:** Health seeking behaviors

	All		Boys		Girls		
	n	% (SE)	n	% (SE)	n	% (SE)	p-value
In the prior 6 months, where did you go for health care services?							*X* ^2^ = 31.0 p < .0001
Health center/hospital/clinic	184	72.5 (2.9)	112	87.7 (3.1)	72	57.8 (4.7)	
Traditional healer/shop keeper/VHSG	69	27.5 (3.0)	16	12.3 (3.1)	53	42.2 (4.7)	
Reason for seeking health services in prior 6 months							-
Prevention screening	38	14.4 (2.6)	24	17.5 (3.0)	14	11.3 (3.2)	
Curative screening	202	78.5 (3.1)	98	73.8 (3.6)	104	83.2 (3.8)	
Sexual reproductive health services	18	6.7 (1.5)	12	8.7 (2.2)	6	4.7 (2.0)	
Treatment for family member	1	0.4 (0.4)	-	-	1	0.9 (0.9)	
Health provider type							*X* ^2^ = 7.3 p = 0.03
Physician/Nurse/Pharmacist	149	56.7 (4.0)	88	64.7 (4.0)	61	48.6 (5.6)	
Village shop keeper/peer counselor	103	39.5 (3.7)	43	31.6 (3.7)	60	47.4 (5.5)	
Traditional healer	10	3.8 (1.3)	5	3.7 (1.5)	5	3.9 (2.0)	

A common reason by the rural youth (78.5 %) for seeking health care services was the need for curative services (i.e. infection, injury or family visit). Other reasons reported were prevention screening (14.4 %) and sexual and reproductive health services (6.7 %). This low percent of youth seeking reproductive health services might be explained by unbelief among youth, that these services do not cater to them. Boys (8.7 %) reported visiting a medical provider for sexual and reproductive health services about twice as much as girls (4.7 %).

The most common healthcare provider type reported was a medical professional such as a physician, nurse, or pharmacist (56.7 %). This was followed by a shop keeper or peer counselor (39.5 %). A small number of youths did visit a traditional healer (3.8 %). More boys visited a medical professional than girls.

### Alcohol use and risky practices

A summary of findings in regards to rural youth alcohol use and risky practices are in Table 4. Self-reported alcohol use in prior 30 days was 27.6 % in rural youths. The frequency of alcohol use between 1–5 times was 89.5 % in youths. A small percent reported using alcohol more than 13 times (7.6 %). More boys used alcohol than girls (74.2 % vs. 57.7 %). We further asked about sex under the influence of alcohol and found that 41.7 % of rural youths reported engaging into sexual activity under the influence. However, there was a significant difference between sexes (p = 0.0006) where girls reported a high percent (77.2 %) relative to boys (29.3 %). In respect to rural youth behavior, we summarize self-reported risky practices in Table 4. The youth reported out late at night with friends (26.5 %), gambling (10.9 %), pornography use (10.9 %), and practicing risky behavior with peers (51.2 %). More boys than girls reported out late at night with friends (41 %), gambling (18.5 %), and pornography use (20.4 %). However, more girls (64.4 %) reported that they practice risky behaviors than boys. Only 5 youths (all males) reported ever trying drugs. No youths reported using a condom while under the influence of alcohol or drugs.

**Table 4:** Alcohol use and youth risky behaviors

	All		Boys		Girls		
	n	% (SE)	n	% (SE)	n	% (SE)	p-value
Youth alcohol use							
Alcohol use in prior 30 days							*X* ^2^ = 22.3, p = <.0001
Yes	74	27.6 (2.1)	56	40.9 (3.7)	18	14.1 (2.9)	
No	189	72.4 (2.3)	80	59.1 (3.9)	109	85.9 (2.8)	
Frequency of alcohol use in prior 30 days							-
Only 1 time	25	34.1 (5.3)	14	25.6 (5.9)	11	57.7 (11.6)	
2 - 5 times	42	55.4 (5.5)	34	60.2 (6.9)	8	57.7 (11.6)	
6 - 12 times	2	2.7 (1.9)	2	3.7 (2.5)	-	-	
13 or more times	6	7.6 (3.6)	6	10.3 (5.0)	-	-	
Number of intoxications in prior 30 days							*X* ^2^ = 13.6, p = 0.001
Only 1 time	30	41.5 (5.6)	16	29.3 (6.3)	14	77.1 (9.2)	
2 - 5 times	41	54.4 (5.5)	38	67.1 (6.2)	3	17.3 (7.8)	
Do not remember	3	3.9 (2.2)	2	3.4 (2.3)	1	5.4 (5.5)	
Sex under influence of alcohol							*X* ^2^ = 14.7, p = 0.0006
Yes	30	41.7 (5.7)	16	29.3 (6.3)	14	77.2 (9.3)	
No	41	54.4 (5.6)	38	67.2 (6.2)	3	17.4 (7.9)	
Don’t remember	3	3.9 (2.2)	2	3.5 (2.4)	1	5.4 (5.6)	
Youth risky behaviors							
Out late at night with friends							*X* ^2^ = 35.8, p < .0001
Yes	71	26.5 (3.2)	56	41.0 (3.9)	15	11.9 (3.9)	
No	192	73.4 (3.1)	80	58.9 (3.9)	112	88.0 (3.8)	
Gambling							*X* ^2^ = 12.2, p = 0.0005
Yes	30	10.9 (1.8)	25	18.5 (3.6)	5	3.9 (1.7)	
No	233	89.0 (1.7)	111	81.5 (3.5)	122	96.1 (1.6)	
Pornography use							*X* ^2^ = 25.1, p = <.0001
Yes	30	10.9 (1.8)	28	20.4 (3.3)	2	1.5 (1.1)	
No	233	89.1 (1.8)	108	79.6 (3.3)	125	98.5 (1.1)	
Practice risky behaviors with peers							*X* ^2^ = 12.3, p = 0.004
Yes	134	51.2 (4.2)	52	38.1 (3.9)	82	64.4 (6.9)	
No	129	48.8 (4.2)	84	61.9 (3.9)	45	35.6 (6.9)	

### Individual and social comparisons

Comparative analysis of individual and social behaviors between sexes is summarized in Table 5. Boys were 4.2 times more likely to consume alcohol relative to girls (OR = 4.2; 95 % CI: 2.3, 7.8). We were unable to perform an analysis of drug use. In regards to social behaviors, boys were 5.2 times more likely to be out late at night with friends relative to girls (OR = 5.2; 95 % CI: 2.8, 9.9). Furthermore, boys were more likely to use pornography (OR = 16.2; 95 % CI: 3.8, 69.6), gamble (OR = 5.5; 95 % CI: 2.0, 14.8), and practice risky behavior with peers (OR = 2.9; 95 % CI: 1.8, 4.9) relative to girls.

**Table 5:** Individual and social comparisons^a^

	Boys	Girls	OR 95 % CI
	n (%)	n (%)	
Individual behaviors			
Alcohol use	56 (40.9)	18 (14.1)	4.2 (2.3, 7.8)
Ever tried drugs	5 (3.6)	-	-
Social behaviors			
Out late at night with friends	24 (25.4)	16 (48.9)	5.2 (2.8, 9.9)
Pornography use	28 (20.4)	2 (1.5)	16.2 (3.8, 69.6)
Gambling	25 (18.5)	5 (3.9)	5.5 (2.0, 14.8)
Practicing risky behaviors^b^ with peers	52 (38.1)	82 (64.4)	2.9 (1.8, 4.9)

^a^Female referent, ^b^includes alcohol and drug use, out late with friends, pornography use

### Logistic regression

In respect to the relation between school enrollment and parental supervision, we report odds ratios on selected rural youth attitudes and risky behaviors in Table 6. We found that current school enrollment had limited impact on the youth attitudes seeking sexual and reproductive health services (adjusted OR = 1.5, 95 % CI: 0.5, 4.6) and feeling ready to cause a pregnancy (adjusted OR = 1.7, 95 % CI: 0.7, 3.9). There were increased odds of both these attitudes in the youth; however, neither of these attitudes were significantly related to school enrollment.

**Table 6:** Effects of school enrollment and living with both parents on youth attitudes and risky behavior

	Currently enrolled in school	
	Crude OR	Adjusted OR^b^
Youth attitude		
Will seek SRH^a^ services	1.1 (0.4, 3.1)	1.5 (0.5, 4.6)
Feel ready to cause pregnancy	2.5 (1.2, 5.2)	1.7 (0.7, 3.9)
Youth risky behavior		
Out late at night with friends	3.5 (2.0, 6.2)	2.2 (1.1, 4.4)
Gambling	1.2 (0.5, 2.6)	0.9 (0.4, 2.3)
Pornography use	2.2 (1.0, 4.7)	1.2 (0.5, 3.1)
Practice risky behaviors with peers	1.2 (0.7, 2.1)	0.78 (0.42, 1.4)
	Living with both parents	
Youth attitude		
Will seek SRH^a^ services	2.9 (1.0, 8.9)	3.1 (1.0, 9.2)
Feel ready to cause pregnancy	0.84 (0.3, 2.5)	0.82 (0.3, 2.5)
Youth risky behavior		
Out late at night with friends	2.7 (0.9, 8.0)	3.3 (1.0, 10.6)
Gambling	1.9 (0.4, 8.4)	1.8 (0.4, 8.5)
Pornography use	4.3 (0.5, 32.8)	4.5 (0.5, 35.9)
Practice risky behaviors with peers	1.8 (0.8, 4.1)	2.0 (0.9, 4.5)

^a^Sexual reproductive health, ^b^Adjusted for age and sex

We examined the association between school enrollment and risky behaviors, and found that gambling, pornography use, and practicing risky behaviors with peers were not significantly associated. Only out late at night with friends was significantly associated with school enrollment (adjusted OR = 2.2, 95 % CI: 1.1, 4.4); however, the effects were negative or at increased odds, suggesting no impact of school enrollment on risky behavior.

Next, we related living with both parents to rural youth attitudes and risky behaviors. We found that living with both parents did increase odds of seeking sexual and reproductive health services (adjusted OR = 3.1, 95 % CI: 3.1, 9.2) but decreased odds of feeling ready to cause a pregnancy (adjusted OR = 0.8, 95 % CI: 0.3, 2.5). Risky behaviors gambling, pornography use and practicing risky behaviors with peers were not significantly associated with living with both parents. Only out late at night with friends was significantly associated with living with both parents. The direction of the association was unexpected and remained after adjustment (OR = 3.3, 95 % CI: 1.0, 10.6).

## Discussion

We report findings of a reproductive health survey of youth in two rural provinces of Cambodia. Rural Cambodian youth, who represent a substantial proportion of the national population, are the nation’s future both economically and socially. We found that rural youths adhered to traditional Cambodian (Buddhist) family values, attitudes and beliefs about sexual health. Although many rural youths initially responded that they would answer questions related to their sexual health, many declined to respond to questions about their personal sexual practices. However, our findings do confirm previous studies, that rural youth do value Cambodian family traditions, values, and beliefs regarding deferment of sexual activity until after marriage [30].

As expected, we found a majority of youths (79.8 %) want to marry. We found that over 90 % of youth reported the value of the ideal for both a boy’s and girl’s deferment of sexual activity until after marriage. Moreover, both boys and girls reported similar values when asked by our interviewer based peer-to-peer interviewer criterion. The data indicate that traditional values are prominent in a sample of rural youth.

Contrary to above findings, we found that a majority of youths (>84 %) plan to have at least one single sexual partner in their remaining adolescent years. However, this may indicate that most youths may not plan premarital intercourse with a boy/girlfriend or partner but rather with a future spouse since many have reported that they value their Khmer cultural practices [31]. Our data supports existing sexual reproductive health programs that are available and supports new intervention programming in rural communities where the youth lack any health information or life-skill training.

Ever had sex was reported by only 14 rural youths (or 5.4 %), which is lower than prior estimates of 12.7 % in high school youths [13]. This highlights the deferment of sexual activity in rural youths that might be influenced by traditional values about marriage and sexuality. Since a majority of youth did not report having sexual intercourse, this may indicate either potential underreporting (since premarital intercourse is discouraged) or an adherence to their traditions. Supporting this preliminary hypothesis is the reported age of sexual debut. We found that boys reported a higher age of sexual debut than girls. Overall, the mean age of sexual debut was about 20 years. Yi et. al. [15] found similar findings in a larger compatible sample and support our findings.

However, we cannot rule out an underreporting of boy’s age of sexual debute since peer pressure from family and friends encouraging boys and discouraging girls from sexual activity. Cambodian family values encouraging a girl’s adherence to chastity more often than a boy’s that suggests a double standard. A sexual encounter prior to marriage for Cambodian boys is sometimes expected prior to their marriage [32].

Finally, we found that a small number of youths (all boys) did report having more than one sexual partner in the prior six months, with five boys reporting that their previous sexual partner was a commercial sex worker. These findings may suggest that although many youth report adherence to their traditional values, this may not be enough to discourage some youths from engaging in risky sexual behaviors.

A portion of rural youths (27.6 %) reported that they had consumed alcohol in the prior 30 days. A prior study by Yi S. et al. [15] reported a higher prevalence (47.4 %) of alcohol use among Cambodian youth. The mean number of alcoholic drinks reported by youths (2–5 drinks) may be indicative of frequent or even regular consumption of alcohol in our sample population. This is disturbing since alcohol is associated with anti-social behavior, increased risk of sexual transmitted diseases, domestic violence, and illicit drug use. Alcohol use has been reported as an antecedent for premarital intercourse and the use of commercial sex workers [13, 33–35]. More alarmingly, the frequency of alcohol use exceeded 13 or more times in a single 30 day period among some of the youth. Moreover, over 41.7 % of drinking youths reported to have sex under the influence of alcohol. This supports the need for culturally based initiatives that prevent and educate rural youths regarding alcohol use and addiction.

Similar to previous studies, relative to girls, boys were more likely to engage in risky behaviors [14, 15]. There might be a double standard in cultural expectations between boys and girls, which prohibits any risky behaviors among girls while allowing risk taking for boys. This cultural norm is very prominent in rural areas of Cambodia where male dominance over the household is frequently encountered. Most disturbing is the use of pornographic products that might influence a boy’s impression of girls and their role in the household. Previous studies indicate that alcohol use is associated with pornography materials [36] and migratory practices [34]. Pornography has been reported to have a severe negative impact on the sexual reproductive health of the youth [37].

Our study highlights some major gender-related issues that predispose boys to less than ideal reproductive health outcomes than girls. The prevailing double standard which allows men to have multiple partners while women are expected to be virgins until they marry places boys at greater risk of contracting sexually transmitted infections [32].

Current school enrollment did not increase odds of youth attitudes of seeking sexual and reproductive health services and feeling ready to cause a pregnancy. Our findings are confirmed by previous studies [13, 38, 39] indicating that a youth’s enrollment in school has limited impact on the likelihood of risky behaviors such as feeling ready to cause a pregnancy which could lead use to a number of negative sexual health outcomes.

Previous studies indicate that a youth’s closeness to adults such as teachers or their parents plays a central role in their lives as they progress through adolescence and into adulthood [13, 40, 41]. In addition, it is reported that among the youth, a teacher may be important in early orientation toward peers [42]. Educational attainment can have a dramatic effect on the life of a youth since health knowledge and life-skills can be acquired by continuous school enrollment that has been associated with positive sexual health outcomes [12].

Current living with both parents did increase odds of going out late at night with friends. These are unexpected findings since previous studies report that youths who have a harmonious relationship with their family are less likely to be associated with delinquent peers and to engage in risky practices [43]. Moreover, parents are important to youth since they provide social norms related to appropriate behavior, as well as having an important to function in the supervision and monitoring of youth’s inclination toward risky behaviors.

We did observed non-significant increased risks of other risky behaviors among rural youths. This might be explained by the reported under preparedness of rural adults in promoting and explaining sexual and reproductive health biology and health issues. Many times the parents of rural youths lack the educational background to explain issues of sexual reproductive health, contraception use, negative health outcomes, and the planning for a family, to their children [12]. However, we did observed an increase in odds of seeking sexual and reproductive health services among the rural youth. This might be explained by alternative sources of sexual and reproductive knowledge such as a teacher or peer counselor. This is noteworthy and encouraging.

### Limitations

The presented study has limitations that warrant some discussion. First, as with any self-reported measures, there are inherent biases that are potential for both underreporting and over reporting. We could not rule out that some youth might by under reporting their sexual practices and risky behaviors. Cambodian culture does not allow free discussion of sexual issues and disapproves of girls engaging in premarital sex while tolerating men’s engaging in premarital sex. This might explain our low response rate. We piloted our questionnaire and in addition reviewed the questionnaire with local public health professionals. However, it might be possible that not all questions were presented in an acceptable form to each youth. There is a potential for a lack of key and culturally appropriate phrases not included in our questionnaire.

Second, our sampling scheme only included rural youths; therefore, our findings may not be generalizable to youths living in urban or semi-urban centers where the lifestyle of such youths might be different. Third, the cross-sectional design did not allow for the assessment of causal relationships between risk factors and sexual and reproductive health indicators in rural youth. Our small study population had twice as many young girls as boys and might have had an effect in sex comparisons of youth attitudes and risky behaviors.

Finally, although we used a multi-stage sampling design, it is still possible that sampling error might explain some of our results. Furthermore, our small sample size limited our evaluation of relationships in sexual and reproductive health characteristics of the rural youth. We were unable to examine several predictors of risky sexual behavior using regression modeling. We were not able to evaluate other reproductive health practices such as contraception (i.e. condom use) due to low response.

## Conclusion

Our study of rural Cambodian youths contributes to previous studies indicating that reproductive health is a public health concern in developing countries such as Cambodia. Although there were positive findings compatible with protective traditional Cambodian values and beliefs, we found youth were challenged with social problems like alcohol use. The reported prevalence of migratory practices might present an opportunity for exposure to risk factors such as HIV infection, occupational abuse, the commercial sex industry, and risky behaviors. Our findings support the current need for sexual and reproductive health prevention in rural areas of Cambodia. This includes the need for school based alcohol and drug use prevention and strengthening or promoting family and community support interventions for young people.

## References

[1] Nations U. World Population Monitoring Adolescents and Youth A Concise Report (2012). New York.

[2] Gubhaju BB. Adolescent Reproductive Health in Asia, in South-East Asia's Population in a Changing Asian Context. Bangkok, Thailand: United Nations Children’s Fund (UNICEF) 2002.

[3] Clayton T (1998). Building the New Cambodia: Educational Destruction and Construction under the Khmer Rouge, 1975–1979. History of Education Quarterly.

[4] Sokhom H (2004). Education in Cambodia: highland minorities in the present context of development. Inter-Asia Cultural Studies.

[5] Cambodia, N.I.o.S (2004). National Institute of Statistics, Cambodia Inter-Censal Survey 2004.

[6] Measure, D. and I. Macro, Cambodia Demographic and Health Survey (2010). 2011: National Institute of Statistics.

[7] Fordham, G., Adolescent and Youth Reproductive Health in Cambodia, in POLICY Project a 13-country study of adolescent reproductive health issues, policies, and programs on behalf of the Asia/Near East Bureau of USAID, K. Hardee, Editor. 2003: Cambodia.

[8] Population Reference Bureau. The world's youth: 2013 data sheet. Population Reference Bureau: www.prb.org.

[9] NIPH, NIS, and ORC Macro, Cambodia Demographic and Health Survey. Phnom Penh, Cambodia: National Institute of Public Health; 2005.

[10] Council, U.P (2009). The adolescent experience in-depth: using data to idenfity and reach the most vulnerable young people Cambodia 2005.

[11] World Health Organization, Sarah Bott (2003). *Towards Adulthood: exploring the sexual and reproductive health of adolescents in South Asia*.

[12] Tan C (2007). Education reforms in Cambodia: issues and concerns. Educational Research for Policy and Practice.

[13] Yi S (2010). Role of risk and protective factors in risky sexual behavior among high school students in Cambodia. BMC Public Health.

[14] Yi S (2011). Risk vs. protective factors for substance use among adolescents in Cambodia. Journal of Substance Use.

[15] Yi S (2014). Factors Associated with Risky Sexual Behavior among Unmarried Most-at-Risk Young People in Cambodia. American Journal of Public Health Research.

[16] United Nations, Situation Analysis of the Youth in Cambodia. 2009, United Nations in Cambodia: Office of the United Nations Resident Coordinator in Cambodia.

[17] Ministry of Planning, C., General Population Census 2008 Royal Kingdom of Cambodia. Phnom Penh Cambodia: Ministry of Planning; 2008.

[18] UNICEF (2015). The State of the World’s Children 2015: Reimagine the Future: Innovation for Every Child.

[19] Chuob SK, P.P. The Future: Youth and HIV/AIDS in Cambodia. in International Conference on AIDS. Phnom Penh Cambodia: Ministry of Planning; 2002.

[20] Chen PF (2008). HIV/AIDS Prevention among Young People in East and South-East Asia in the Context of Reproductive and Sexual Health. Asia-Pacific Population Journal.

[21] Tarr CM, Aggleton P (1999). Young people and HIV in Cambodia: Meanings, contexts and sexual cultures. AIDS Care.

[22] Ministry of Education Cambodia, M.o. Education (2004). *National Survey on Youth Risk Behavior (Age 11–18) 2004*.

[23] UNICEF and UNESCO. 2004 Cambodian National Youth Risk Behavior Survey. Ministry of Education Editor. 2004. Phenom Phen: p. 1–76.

[24] Tylee A (2007). Youth-friendly primary-care services: how are we doing and what more needs to be done?. The Lancet.

[25] Kesterton AJ, de Mello MC (2010). Generating demand and community support for sexual and reproductive health services for young people: A review of the Literature and Programs. Reprod Health.

[26] Bearinger LH (2007). Global perspectives on the sexual and reproductive health of adolescents: patterns, prevention, and potential. The Lancet.

[27] ADRA Cambodia and Nyirady Stephen (2009). Rural Cambodian Youth Sexual Reproductive Health Project.

[28] Ministry of Health Cambodia, *Ministry of Health National Ethics Committee for Health Research Standard Operating Procedures*, M.o. Health, editor. 2008. MOH: Phenom Phen.

[29] PRH, M.E. Family Planning and Reproductive Health Indicators Database. 2015; Available from: http://www.cpc.unc.edu/measure/prh/rh_indicators/specific/arh.

[30] Le TN, Kato T (2006). The role of peer, parent, and culture in risky sexual behavior for Cambodian and Lao/Mien adolescents. Journal of Adolescent Health.

[31] Yang Y, Lewis FM, Kraushaar DL (2013). HIV transmission from husbands to wives in Cambodia: a systematic review of the literature. Cult Health Sex.

[32] Douthwaite M, Sareoun L (2006). Sexual behaviour and condom use among unmarried young men in Cambodia. AIDS Care.

[33] Kebede D (2005). Khat and alcohol use and risky sex behaviour among in-school and out-of-school youth in Ethiopia. BMC Public Health.

[34] Nishigaya K (2002). Female Garment Factory Workers in Cambodia: Migration, Sex Work and HIV/AIDS. Women Health.

[35] Tapert SF (2001). Adolescent substance use and sexual risk-taking behavior. Journal of Adolescent Health.

[36] World Vision Cambodia and Fordham Graham (2006). As if they are watching my body: Pornography and the devleopment of attitudes towards sex and sexual behavior among Cambodian Youth.

[37] Flood M (2009). The harms of pornography exposure among children and young people. Child Abuse Review.

[38] Chiao C, Yi C-C (2011). Adolescent premarital sex and health outcomes among Taiwanese youth: perception of best friends' sexual behavior and the contextual effect. AIDS Care.

[39] Carter M (2007). Health outcomes in adolescence: Associations with family, friends and school engagement. J Adolesc.

[40] Rice F, A.a. Bacon (1999). *The Adolescent: Development, relationship, and culture*.

[41] Scales PC (1999). Reducing risks and building developmental assets: essential actions for promoting adolescent health. J Sch Health.

[42] O'Donnell CR (2003). Culture, Peers, and Delinquency. J Prev Interv Community.

[43] Werner NE, Silbereisen RK (2003). Family Relationship Quality and Contact with Deviant Peers as Predictors of Adolescent Problem Behaviors: The Moderating Role of Gender. Journal of Adolescent Research.

